# Regulatory Mechanism for Absence Seizures in Bidirectional Interactive Thalamocortical Model via Different Targeted Therapy Schemes

**DOI:** 10.1155/2021/1198072

**Published:** 2021-09-16

**Authors:** Hudong Zhang, Xiaolong Tan, Yufeng Pan, Yuan Chai

**Affiliations:** School of Mathematics and Physics, Shanghai University of Electric Power, Shanghai 201306, China

## Abstract

Recent clinical practice has found that the spike-wave discharge (SWD) scopes of absence seizures change from small cortical region to large thalamocortical networks, which has also been proved by theoretical simulation. The best biophysics explanation is that there are interactions between coupled cortico-thalamic and thalamocortical circuits. To agree with experiment results and describe the phenomena better, we constructed a coupled thalamocortical model with bidirectional channel (CTMBC) to account for the causes of absence seizures which are connected by the principle of two-way communication of neural pathways. By adjusting the coupling strength of bidirectional pathways, the spike-wave discharges are reproduced. Regulatory mechanism for absence seizures is further applied to CTMBC via four different targeted therapy schemes, such as deep brain stimulation (DBS), charge-balanced biphasic pulse (CBBP), coordinated reset stimulation (CRS) 1 : 0, and (CRS) 3 : 2. The new CTMBC model shows that neurodiversity in bidirectional interactive channel could supply theory reference for the bidirectional communication mode of thalamocortical networks and the hypothesis validation of pathogenesis.

## 1. Introduction

Absence seizures characterized by spike-wave activity were first discovered in 1941 by the electroencephalograms (EEGs) of patients [1], whose frequency is shown at a range of approximately 2–4 Hz [2]. The classic symptoms of seizures are anxiety and depression compromising quality of patient life. Absence seizures are particularly prevalent among young people [3]. It is a heavy affliction tortured by clouding of consciousness and temporary disturbance of consciousness when patients are at the onset of a seizure [4, 5]. Complex brain dynamics [6, 7] may cause the rhythmic spike-wave discharges of neurological disorders due to the exceptional transmissions between cerebral cortex and thalamus [8, 9]. Recent theoretical researches on this neurological disorder have also confirmed the above points in the coupled thalamocortical model and drilled down to deeper levels of induced mechanism of SWD [10–12].

In the aspect of modeling, the spatially extended neural field model has become a hot area of research in recent years [13–15]. The model embodies the dynamics of cerebral cortex incorporating stereotactic space and distance [16–18]. Due to their coupling interactions with each other, dynamical behaviors of firing neurons can be reproduced, which can be regarded as a reference model of macroscopic absence seizures in epilepsy. The original model containing four neurons in the space of brain has been proposed in [19]. The coupled thalamocortical model consisting of two different coupled compartments with short-range connection has been reported in [20]. A basal ganglia corticothalamic (BG-CT) model has been expanded via replacing basal ganglia by a 2I:3O feedback modulator [21]. Until now, the spatiotemporal characteristics have not been fully exploited. Therefore, it is necessary to construct a theoretical model to explore a wide range of pathogenic possibilities. However, it remains uncertain whether or not absence seizures exist in coupled thalamocortical model with interactional channel.

In the aspect of neurostimulation, countries in the whole world are making substantial progress in seeking access to epilepsy seizure therapies [22–24]. For patients with drug-resistant epilepsy [25, 26], deep brain stimulation (DBS) [27–29] and coordinated reset stimulation (CRS) [30–32] are two control schemes most widely used in the treatment of neurological disorders. Both candidates have their pros and cons in controlling energy consumption and side effects: the advantage of DBS itself is that epileptic circuitry is effectively blocked under successive strong stimuli against focus areas, while the successive strong stimuli are highly energy consuming and tissue-destructive; the admirable point of CRS is that multiple parts of the brain can be added brief pulse trains with small side effects despite the drawback of being slow. Subsequently, a new of treatment scheme named periodic charge-balanced biphasic pulse (CBBP) is proposed by combining both control schemes above [33, 34]. It is valuable to test which therapeutic stimulation plan is preponderant in achieving the best therapeutic effect [35–38], although it is still an open question as for the optimal scheme of improving cure rates and reducing CTMBC risks. Therefore, it is necessary to select a neurostimulation with less side effect and energy consumption.

To sum up, a coupled thalamocortical network evolved from a neural field model is composed mainly of four neuronal populations with unidirectional information transfer. Inspired by these excellent results, a large amount of unknown space is found in coupled thalamocortical model with interactional channel [39, 40]. To reveal bidirectional interactive transmit, an extended theoretical model should be established to investigate the mechanism of induced epilepsy seizures in interactional channel macroscopically. Therefore, to further show multichannel transmission, a coupled bidirectional cortico-thalamic model constituted by eight neuronal populations with unidirectional connection structure and is expanded from the coupled cortico-thalamic model. Here, we address the above unsolved issues on the previous classical coupled models. Peculiarly, we develop a coupled cortico-thalamic model by viewing unidirectional channel as a bidirectional channel. The CTMBC led to the occurrence of absence seizures induced by interactional channel and the discovery of focal area of epilepsy. Corresponsively, four targeted therapy schemes including DBS, CBBP, CRS1:0, and CRS3:2 are added to the focal area to resist epilepsy.

In this paper, we focus on the regulatory mechanism of coupled thalamocortical model with bidirectional interactive channel. The next section describes the coupled thalamocortical model and four therapeutic plans in detail, including DBS, CBBP, CRS 1 : 0, and CRS 3 : 2. The absence seizures under double-directional transmission and the optimal treatment plan are obtained in Section 3. Finally, the results of modeling and simulation are presented in Section 4.

## 2. The Principle of Connection and Schemes of Treatment

**Figure 1:**
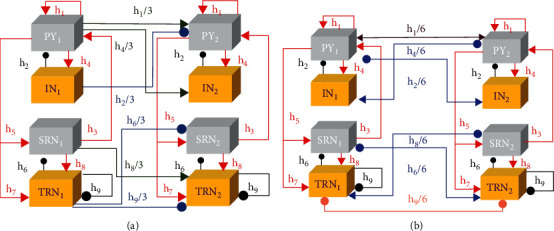
Layout diagrams of the coupled thalamocortical model (CTM) without and with bidirectional channel. (a) Original CTM consists of two regions of PY–IN cortex and SRN–TRN subcortical circuit [20]. (b) Coupled thalamocortical model with bidirectional channel (CTMBC). The arrow and round headlines represent the excitatory and inhibitory projections from glutamate and GABAA receptors, respectively. The midrange and bidirectional connections are adopted in cortex and subcortical circuit between two different coupled modules. Double arrow lines and double round headlines denote the same bidirectional excitatory and inhibitory projections, respectively. Single arrow lines and single round headlines at the opposite ends of the projections denote different interactional channels (color figure online).

There are four types of neuronal populations shown in the original Taylor model [19], which are comprised of thalamic reticular nucleus (TRN) and specific relay nucleus (SRN) in the subcortical pathway and inhibitory interneuronal population (IN) and pyramidal neuronal population (PY) in the cortex, where pathological SWD activity is reproduced and a single pulse stimulation is given to control epileptic seizures. To explore how the cortico-cortical connectivities affected different macroscopic dynamical phenomena, some modified models were extended in spatial pathways [17, 18, 20, 21]. Because different organizations transfer in different ways, the unidirectional connection from the thalamus to the cerebral cortex between module I and module II was not considered, which is shown in Figure 1(a). The extended model composed by two coupled module circuits can reveal SWD oscillation of epilepsy. The arrow and round headlines represent the excitatory and inhibitory projections from glutamate and GABAA receptors, respectively. The midrange and bidirectional connections are adopted in cortex and subcortical circuits between two different coupled modules.

In order to explore the disease mechanism of absence seizures, we construct a bidirectional channel model to simulate the neural kinetic processes in coupled cortico-thalamic thalamocortical circuits, namely, coupled module I and module II. The schematic of coupled model with bidirectional channel has been displayed in Figure 1(b). Double arrow lines and double round headlines denote the same bidirectional excitatory and inhibitory projections, respectively. Single arrow lines and single round lines at the opposite ends of the projections denote different interactional channels. The coupled model has two modules, consisting of a cerebral cortex and a subcortical circuit, which systematically divide the internal space into eight populations, i.e., PY_*i*_ and IN_*i*_ in the cerebral circuits and SRN_*i*_ and TRN_*i*_ in the subcortical circuits (*i* = 1, 2). There are two main forms of interneuronal population: excitatory and inhibitory. The former originates from SRN_*i*_ and PY_*i*_, and the latter TRN_*i*_ and IN_*i*_ (*i* = 1, 2). Our modified model establishes a two-module coupled cortico-thalamic network with bidirectional path to explore absence seizures and macroscopic nonlinear kinetics characteristics. The set of coupled model breaks up into four equations with one set of two simultaneous equations in each, defined as shown below:

(1) $$\begin{array}{c} \left\{\begin{array}{c} \frac{{\text{dPY}}_{1}}{\text{dt}}=\left(\varepsilon_{py}-{\text{PY}}_{1}+h_{1}Q\left[{\text{PY}}_{1}\right]-h_{2}Q\left[{\text{IN}}_{1}\right]+h_{3}Q\left[{\text{SRN}}_{1}\right]\right)\tau_{1}+\frac{h_{1}}{6}Q\left[{\text{PY}}_{2}\right]-\frac{h_{2}}{6}Q\left[{\text{IN}}_{2}\right],\\ \frac{{\text{dPY}}_{2}}{\text{dt}}=\left(\varepsilon_{py}-{\text{PY}}_{2}+h_{1}Q\left[{\text{PY}}_{2}\right]-h_{2}Q\left[{\text{IN}}_{2}\right]+h_{3}Q\left[{\text{SRN}}_{2}\right]\right)\tau_{1}+\frac{h_{1}}{6}Q\left[{\text{PY}}_{1}\right]-\frac{h_{2}}{6}Q\left[{\text{IN}}_{1}\right], \end{array}\right. \end{array}$$

(2) $$\begin{array}{c} \left\{\begin{array}{c} \frac{{\text{dIN}}_{1}}{\text{dt}}=\left(\varepsilon_{\text{in}}-{\text{IN}}_{1}+h_{4}Q\left[{\text{PY}}_{1}\right]\right)\tau_{2}+\frac{h_{4}}{6}Q\left[{\text{PY}}_{2}\right],\\ \frac{{\text{dIN}}_{2}}{\text{dt}}=\left(\varepsilon_{\text{in}}-{\text{IN}}_{2}+h_{4}Q\left[{\text{PY}}_{2}\right]\right)\tau_{2}+\frac{h_{4}}{6}Q\left[{\text{PY}}_{1}\right], \end{array}\right. \end{array}$$

(3) $$\begin{array}{c} \left\{\begin{array}{c} \frac{{\text{dSRN}}_{1}}{\text{dt}}=\left(\varepsilon_{srn}-{\text{SRN}}_{1}+h_{5}Q\left[{\text{PY}}_{1}\right]-h_{6}K\left[{\text{TRN}}_{1}\right]\right)\tau_{3}-\frac{h_{6}}{6}K\left[{\text{TRN}}_{2}\right],\\ \frac{{\text{dSRN}}_{2}}{\text{dt}}=\left(\varepsilon_{srn}-{\text{SRN}}_{2}+h_{5}Q\left[{\text{PY}}_{2}\right]-h_{6}K\left[{\text{TRN}}_{2}\right]\right)\tau_{3}-\frac{h_{6}}{6}K\left[{\text{TRN}}_{1}\right], \end{array}\right. \end{array}$$

(4) $$\begin{array}{c} \left\{\begin{array}{c} \frac{{\text{dTRN}}_{1}}{\text{dt}}=\left(\varepsilon_{trn}-{\text{TRN}}_{1}+h_{7}Q\left[{\text{PY}}_{1}\right]+h_{8}K\left[{\text{SRN}}_{1}\right]-h_{9}K\left[{\text{TRN}}_{1}\right]\right)\tau_{4}+\frac{h_{8}}{6}K\left[{\text{SRN}}_{2}\right]-\frac{h_{9}}{6}K\left[{\text{TRN}}_{2}\right]+\beta_{1}\left(t\right)u\left(t\right),\\ \frac{{\text{dTRN}}_{2}}{\text{dt}}=\left(\varepsilon_{trn}-{\text{TRN}}_{2}+h_{7}Q\left[{\text{PY}}_{2}\right]+h_{8}K\left[{\text{SRN}}_{2}\right]-h_{9}K\left[{\text{TRN}}_{2}\right]\right)\tau_{4}+\frac{h_{8}}{6}K\left[{\text{SRN}}_{1}\right]-\frac{h_{9}}{6}K\left[{\text{TRN}}_{1}\right]+\beta_{2}\left(t\right)u\left(t\right). \end{array}\right. \end{array}$$

In the coupled module I and module II, there are four excitatory projections including pyramidal neuronal PY1, PY2 from the cerebral cortex and specific relay nucleus SRN1, and SRN2 from the thalamus. Analogously, there are four inhibitory projections including interneuronal IN1, IN2 from the cerebral cortex and thalamic reticular nucleus TRN1, and TRN2 from the thalamus. The eight populations pass the transaction information to each other by the coupling strengths *h*_1,2,⋯,9_. *τ*_1,2,3,4_ are time scale coefficients, where *h*_1,2,⋯,9_ = (1.8, 1.5, 1, 4, 3, 0.6, [1.5, 2.5], [9, 11], 0.2), *ε*_*py*,in,*srn*,*trn*_ = (−0.35, −3.4, −2, −5), and *τ*_1,2,3,4_ = (26, 32.5, 2.6, 2.6).

The equations *Q* [.] and *K* [.] defined as follows are activation factors [22]:

(5) $$\begin{array}{c} \left\{\begin{array}{c} Q\left[x\right]=\frac{1}{\left(1+\varepsilon^{-x}\right)},\\ K\left[y\right]=ay+b, \end{array}\right. \end{array}$$

where *x* = PY_*i*_ (*i* = 1, 2), IN_*i*_ (*i* = 1, 2), SRN_*i*_ (*i* = 1, 2), and TRN_*i*_ (*i* = 1, 2), and *y* = SRN_*i*_ (*i* = 1, 2) and TRN_*i*_ (*i* = 1, 2). The *b* is a constant. The parameters *ε* and *a* mean the steepness of two activation functions, where *a* = 2.8, *b* = 0.5, and *ε* = 250000 in this paper, respectively. The axons of different neuronal populations have different radiation ranges, which can be roughly classified into three categories: short-range transmission, long-range transmission, and distant excitatory transmission. Three kinds of coupling strength are *h*_*i*_/3, *h*_*i*_/6, and *h*_*i*_/9 in turn. In the CTMBC, there are two coupled modules, eight neuronal populations, and six two-way interactional channels connecting module I and module II. The intermodule coupling strength of long-range transmission is *h*_*i*_/6 to ensure effective connection in the same cerebral cortex areas and thalamus areas. In the cerebral cortex areas of module I and module II, *h*_1_/6 is a bidirectional excitatory projection, and *h*_2_/6 and *h*_4_/6 are bidirectional excitatory-inhibitory projections. In the thalamus areas of module I and module II, *h*_6_/6 and *h*_8_/6 are bidirectional excitatory-inhibitory projections, and *h*_9_/6 is a bidirectional inhibitory projection.

The proposed model is composed of 16 nerve neurons, which are functionally connected to each other and coupled to other parts of the brain. Generally, the above three transmissions are the three main ways to connect coupled compartments whose transmission conditions are quite complex and transmission ways are many and varied. The short-range connection *h*/3 has been widely studied, and its dynamic properties have been given in previous studies, while the dynamic properties of long-range connection *h*/6 are unknown. In particular, the connection distance is relatively far in the process of neuronal interaction. In order to connect coupled adjacent areas, the axons of neuronal population should long enough to affect the distant neurons. We just consider long-range connection *h*/6 described as connection strength which can affect cerebral cortex and thalamus between two coupled compartments, respectively. Therefore, researching the coupled thalamocortical model with bidirectional channels by long-range connection *h*/6 has higher theory value and practical significance.

**Figure 2:**
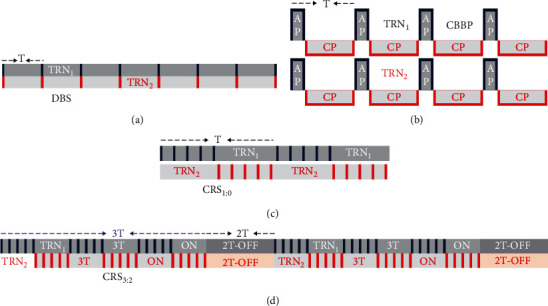
Sketch of four types of therapeutic schemes: (a) deep brain stimulation (DBS), (b) asymmetrical CBBP with no interphase gap, (c) CRS 1 : 0 strategy, and (d) CRS 3 : 2 strategy, respectively (color figure online).

During the process of calculation, a sigmoid activation term located the thalamic circuit *Q*[*x*] = 1/(1 + *ε*^−*x*^) can be approximated by the linear type *K* [*y*] = *ay* + *b*. It is shown that the approximation is available and that the linear range result is in agreement with theoretical one tested by Taylor et al. [19]. Multiple control schemes shown in Figure 2 are added for the treatment of SWD. *u*(*t*) represents the DBS therapeutic plan, and ICRS (*t*) represents the CRS therapeutic plan. The main difference between the control strategies of DBS and CRS 1 : 0 is that the former simultaneously exert stimulation to TRN_*i*_ in the same thalamus areas of module I and module II, and the latter alternately exert stimulation to TRN_*i*_, beginning with TRN_1_. The control strategy of CRS 3 : 2 is an upgraded and controllable schemes compared to CRS 1 : 0. Alternate property beginning with TRN_1_ remains unchanged, but controllable property changes from nonstop alternate stimulation to on-off alternate stimulation, alternately spending stimulating TRN_1_ and TRN_2_ for three cycles and stopping stimulating for two cycles.

In the DBS therapeutic plan, a periodic step function is the principle of operation DBS described as follows [28] (see Figure 2(a)):

(6) $$\begin{array}{c} u\left(t\right)=\alpha \times H\left(\sin\left(\frac{2\pi }{\rho }\right)\right)\left(1-H\left(\sin\left(\frac{2\pi \left(t+\delta \right)}{\rho }\right)\right)\right), \end{array}$$

where an effective stimulus duration *δ* is a positive input pulse; parameters*α* and *ρ* are the stimulation amplitude and period; *H* denotes Heaviside step function. The value of instantaneous frequency *f* is 1/*ρ*. To be effective in reducing the risk of absence seizures, by a contrastive analysis, suitable values of stimulation amplitude, frequency, and positive input pulse for the treatment are selected as *α* = 2 mA, *f* = 130 Hz, and *δ* = 4 ms, respectively.

The CBBP therapeutic plan has anodic pulse (AP), cathodic pulse (CP), and a rectangular waveform with adjustable duration, described as follows [34] (see Figure 2(b)):

(7) $$\begin{array}{c} u_{\text{CBBP}}\left(t\right)=\left\{\begin{array}{cc} \delta , & PT\leq \left|t\right|\leq PT+\delta ,\\ \frac{-\delta }{T-\delta }, & PT+\delta \leq \left|t\right|\leq PT+T, \end{array}\right. \end{array}$$

where *T* and *δ* are the period and duration of pulse current, *P* ∈ *N*.

In the ICRS (*t*) therapeutic plan, the *m* : *n* ON–OFF CRS signal added to TRN_*i*_ (*i* = 1, 2) can be expressed as follows [32] (see Figures 2(c) and 2(d)):

(8) $$\begin{array}{c} I_{\text{CRS}}\left(t\right)=\beta_{1}\left(t\right)u\left(t\right)+\beta_{2}\left(t\right)u\left(t\right), \end{array}$$

where stimulation microelectrodes *β*_1_(*t*) and *β*_2_(*t*) are the stimulus functions. *β*_1,2_(*t*) = 1 and 0 mean start stimulation and end stimulation to epileptogenic focus TRN_1_ and TRN_2_.

In order to obtain the optimal therapeutic plan, the judgment criteria on cure rates and energy consumption should be considered after adding stimulation. In search of an optimal treatment way round the SWD problem, four different stimulation strategies are added to epileptogenic focus. A set of evaluation indices is adopted to compare the advantages and disadvantages of the four strategies, especially indices on the percentage reduction in the number of absence seizures and energy consumption. The root mean square (RMS) is considered to calculate the electrical current stimuli values of ICRS (*t*) and *u* (*t*) defined as follows [20]:

(9) $$\begin{array}{c} I_{\text{RMS}}\left(t\right)=\frac{1}{\sqrt{N}}{\left\|•\right\|}_{2},\left(•=u\left(t\right),I_{\text{CRS}}\left(t\right),u_{\text{CBBP}}\left(t\right)\right), \end{array}$$

where ‖•‖_2_ represents the two norms of the currents *u*(*t*), *I*_CRS_(*t*), and *u*_CBBP_(*t*), respectively. *N* is total time steps.

Most of the parameters used in the CTMBC are in consistency with that of the original experimental studies. The long-range transmission parameters of six two-way interactional channels connecting module I and module II to ensure effective connection in the same cerebral cortex areas and thalamus areas are estimated in numerical studies. Compared to previous studies, the complexity of the model is that the corticothalamic equation set of module I has more coupled terms which are the feedback from module II. Based on the existing results, the coupled thalamocortical model with bidirectional channel is studied deeply in the paper by means of bifurcation simulation, state evolution and frequency analysis, and practical comparison calculating in a relatively simple manner but enough to show the validity and innovation of the model. All simulations are performed up to 30 seconds and the data values from 10 to 30 seconds are employed for statistic analysis. For each numerical setting, 20 independent simulations with different random initial values are carried out to obtain true results, and the averaged result is presented as the final result in the paper. The dynamical differential equations of the CTMBC are solved via the standard fourth-order Runge–Kutta method. All the numerical calculations in the paper are verified in the MATLAB R2019a (MathWorks, USA) simulation environment. All the temporal resolution of numerical integration is 0.25 ms. The integration step is 0.25.

## 3. Numerical Results

### 3.1. TRN Activation Regulating State Transitions

**Figure 3:**
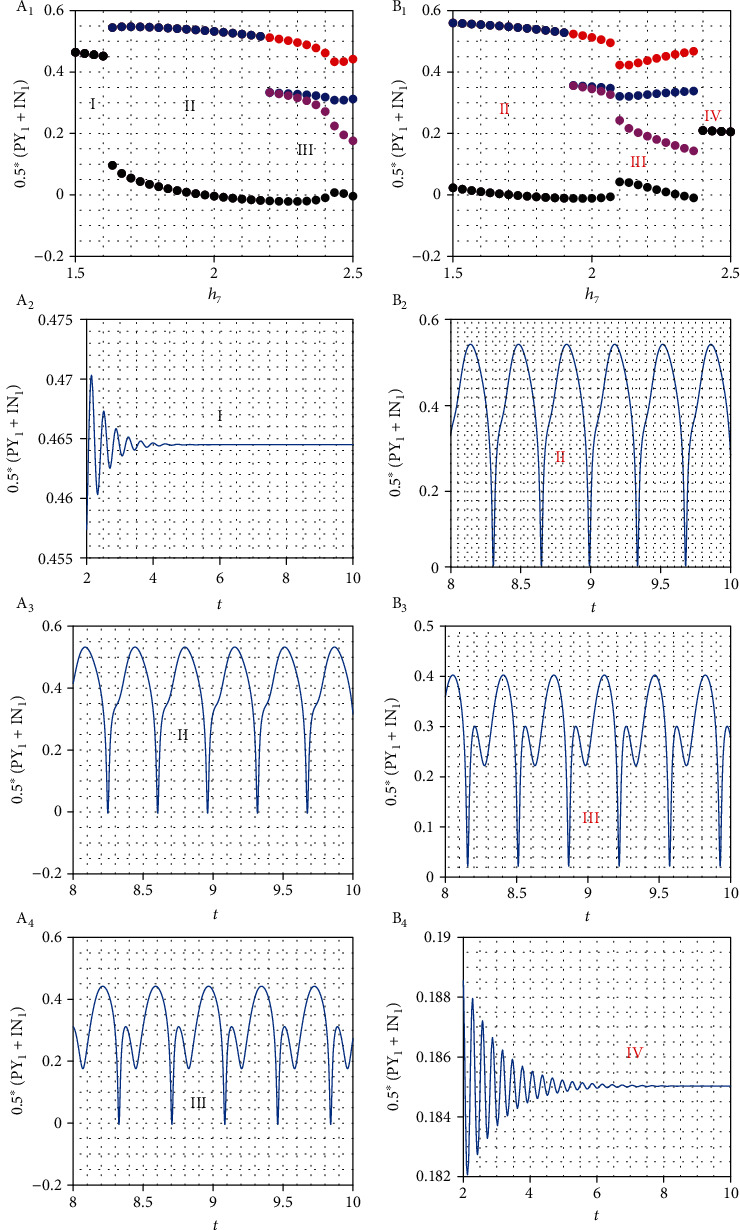
One-dimensional bifurcation diagram. (a) Three dynamical state areas including high saturated state (I), the simple oscillation state (II), and the SWD oscillation state (III). The time series of 0.5(PY_1_ + IN_1_) with different *h*_7_ are shown in (b–d), respectively. (b–d) *h*_7_ = (1.5, 2.1, 2.5) and *h*_8_ = 9.2 are set. (e) Three dynamical state areas including the simple oscillation state (II), the SWD oscillation state (III), and the low firing state (IV). The dynamical evolution of the above three dynamical states are shown in (f–h), respectively. (f–h) *h*_7_ = (1.5, 2.1, 2.5) and *h*_8_ = 10.2 are selected (color figure online).

Previous studies have confirmed that the TRN is core cell relating to the firing of absence seizures both in a single corticothalamic model and coupled model [19–21]. To know whether the same principle also exists in the extended CTMBC, bifurcation analysis for the two coupling strengths *h*_7_ and *h*_8_ is shown, respectively. Above numerous results were gathered to establish proven findings that TRN activation induces absence seizures. Therefore, it is necessary to analyze the relationship between TRN activation and caused absence seizures in our coupled thalamocortical model with bidirectional channel. The TRN activation is closely correlated with two nerve excitatory pathways, PY–TRN pathway and SRN–TRN pathway, marked *h*_7_ and *h*_8_, respectively. To explore the transitions between different states, the bifurcation pattern of cerebral cortex is plotted by changing with two excitatory pathways *h*_7_ and *h*_8_ (see Figures 3(a) and 3(e)), respectively. In reality, EEG data is taken from the firing activities of cerebral cortex consisting of excitatory pyramidal neuronal (PY) population and inhibitory interneuronal (IN) population. Therefore, the mean field potential 0.5(PY + IN) of superimposing above two populations is practical analysis. In our CTMBC, 0.5(PY_1_ + IN_1_) in module I is selected as the main focus to show different dynamical states (see Figures 3(b–d) and 3(f–h)) by bifurcation analysis.

Increasing the coupling strengths, *h*_7_ and *h*_8_ have a double effect on promoting TRN_1_ activation. In order to display this characteristic, the bifurcation analysis of 0.5(PY_1_ + IN_1_) as a function of *h*_7_ is presented from two directions, low *h*_8_ coupling strength and high *h*_8_ coupling strength. In the low value *h*_8_ = 9.2 case, when the coupling strengths *h*_7_ and *h*_8_ are small, the TRN_1_ activation is very low to suppress SRN_1_ activation leading to excitatory firing applied to the cerebral cortex inducing the high saturated state. With the strength *h*_7_ increased, more and more inhibitory neurons from TRN_1_ to SRN_1_ caused SRN_1_ activation decreasing the firing of cerebral cortex. The firing state changes from the high saturated state to the simple oscillation state. Finally, with further increase of coupling strength *h*_7_, the TRN_1_ activation is strong enough to suppress the firing of SRN_1_, which can lead to the occurrence of absence seizures also known as SWD. The bifurcation analysis shows three states: lowing firing state, simple oscillation state, and SWD oscillation state. In the high value *h*_8_ = 10.2 case, there are three state transitions compared to the low value *h*_8_ = 9.2 case under the same the variation regions of *h*_7_, where the high saturated state changes to the simple oscillation state, the simple oscillation state changes to the SWD, and the SWD changes to the lowing firing state. The three transitions illustrate that the larger the *h*_8_ coupling strength, the more active the TRN_1_ activation is. In other words, remaining *h*_7_ original range and increasing *h*_8_ can strengthen TRN_1_ activation working better for the regulation and control of absence seizures.

**Figure 4:**
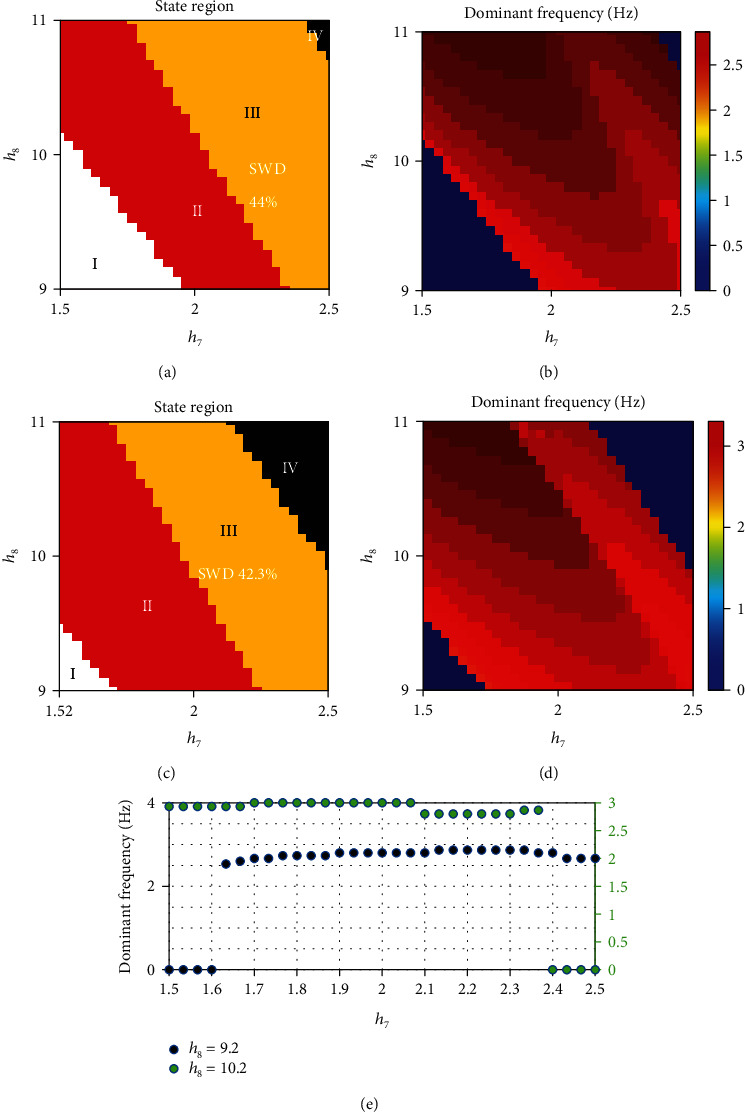
Two-dimensional dynamic analysis in (*h*_7_, *h*_8_) plane and their corresponding dominant frequency. (a, c) State region and (b, d) frequency analysis. I: high saturated state; II: simple oscillation state; III: 2–4 Hz spike and wave discharges (SWD); IV: low firing. (e) The lower panel shows dominant frequency from I to III when *h*_8_ = 9.2 and from II to IV when *h*_8_ = 10.2, respectively (color figure online).

There are two important characteristics in the original coupled model: short-range transmission and unidirectional connection configurations.  Figure 4 displays the comparison effect under two different conditions when the transmission strength is *h*/6 and the connection way is unidirectional, and the transmission strength is *h*/6, and the connection way is bidirectional, which show the advantage of design in selecting internal configuration properties. When changing the short-range transmission and keeping unidirectional connection configurations unchanged, one more discharge state was found in state evolution after changing the short-range transmission to *h*/6. When changing two characteristics that are the short-range transmission to long-range transmission and unidirectional connection configurations to bidirectional connection configurations, compared with changing the characteristic of transmission (Figure 4(a)), state diagram shown in Figure 4(c) has a smaller area of SWD than that shown in Figure 4(a). Therefore, by comparison, the main trends of the coupled thalamocortical model were forwarded to long-range transmission and bidirectional connection configurations due to four EEG activities and a smaller area of SWD in the brain.

The change of short-range transmission to long-range transmission can modify the activation level of neurons, which affects state transition. Indeed, we find that the activation level of SRN_1_ is doubly activated by not only the TRN_1_ but also the TRN_2_, through the *h*_7_ and *h*_8_ and *h*_6_/6 pathways. Further, changing the connection configurations can enhance the activation level of SRN_1_ to narrow down the SWD oscillation areas. Meanwhile, the feedback effect of TRN_2_ shown in Figures 1(b) reveals that, for two fixed PY_1_-TRN_1_ and SRN_1_-TRN_1_ pathways, the SWD area of the coupled model within the 2–4 Hz can be shrunk by the coupled feedback of TRN_2_-SRN_1_ from module II (Figure 4(c)). In especial, owing to the activation of SRN_1_ is related to the growth of *h*_6_/6 pathway, the novel results further indicate that the model exists the better inhibitory effect due to the bidirectional connection configurations, such as 44% SWD area with unidirectional connections (Figure 4(a)) and 42.3% SWD area with bidirectional connections (Figure 4(c)).

From the above discussion, it can be confirmed that the pathways *h*_7_ and *h*_8_ can regulate absence seizures. In strong *h*_7_ region, the SWD suppression is presented by increasing the excitatory pathway SRN–TRN *h*_8_, suggesting that high active TRN neurons may force seizure termination through the TRN–SRN pathway (see Figure 4(c)). In the strong region of *h*_7_ and *h*_8_, such suppression effect is pretty obvious that powerful TRN activation can kick the cortex dynamic state into the low firing region (see Figure 4(c) IV). To further study the combined effect of two excitatory pathways on the regulation of absence seizures, the two-dimensional state and dominant frequency analysis are the best way to show different state regions and corresponding frequencies. In (*h*_7_, *h*_8_) plane, four different state areas are described by different colors in Figures 4(c) and 4(d), which are consistent with the states shown in Figures 3(a) and 3(e). The four different state areas I to IV are filled with four kinds of color, white high saturated state I, red state simple oscillation state II, yellow 2–4 Hz SWD III, and black low firing IV. In particular, the yellow area III whose domain frequency comes within the 2–4 Hz represents SWD oscillation symbolizing the appearance of absence seizures. In general, TRN_1_ activation is a main clue passing through the four different states [41, 42]. Corresponding dominant frequency is displayed by double coordinates in Figures 4(e) and 4(i) to III when *h*_8_ = 9.2 and II to IV when *h*_8_ = 10.2, respectively. The best explanation is that the appearance of four oscillation states in cortical neuronal populations adjusted by inhibitory SRN_1_ transmission affected by GABAA receptors from TRN_1_ which is gradually activated by *h*_7_ and *h*_8_. State evolution is influenced by the increasing value of (*h*_7_, *h*_8_). When the new coupled thalamocortical model is with bidirectional channel, four state transitions reflect more pathological regions being better for clinical detection and treatment.

### 3.2. The Therapeutic Effects of Different Stimulation Strategies

**Figure 5:**
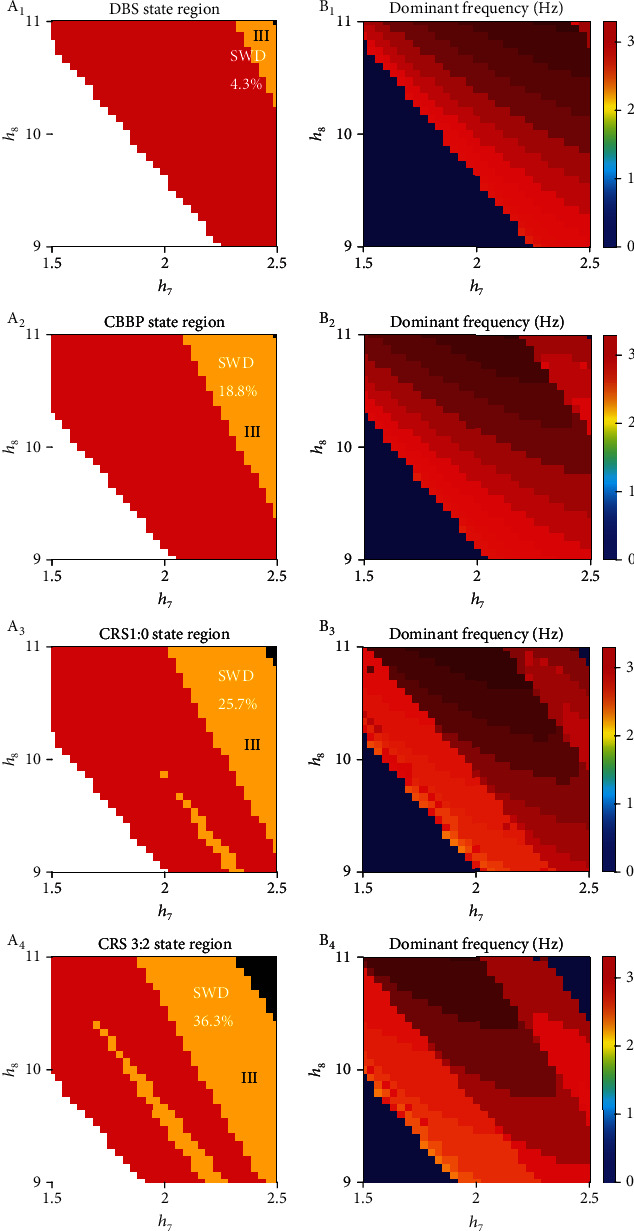
Therapeutic effect diagrams among four different stimulations, respectively. Stimulation parameters are amplitude *α* = 3 mA, the positive input pulse *δ* = 0.004 s, and frequency *f* = 130 Hz. (a, e) Dynamical evolution and corresponding frequency in (*h*_7_, *h*_8_) plane through adding DBS, (b, f) through adding CBBP, (c, g) through adding CRS 1 : 0, and (d, h) through adding CRS 3 : 2. The four yellow regions III signify the size of absence seizures in (*h*_7_, *h*_8_) plane (color figure online).

In therapeutic effects, surgical in resection of epileptogenic foci is seldomly used owing to higher risks and severe trauma. Medication treatment for epilepsy is not up to expected effect and has side effect in a certain extent. Therefore, the electrical nerve stimulation of DBS, CBBP, and CRS became the main approaches in treatments for epilepsy. In our model, there are TRN_1_ and TRN_2_ two cores induced epilepsy. However, it is still unknown which stimulation would help patients to realize reducing disease, symptoms, and spread. In this section, we apply four different stimulation plans to TRN populations to evaluate the effect of the treatments in seizure inhibition. The detailed strategies of DBS, CBBP, CRS 1 : 0, and CRS 3 : 2 are also displayed in Figures 2(a)–2(d). The size of SWD area in two-dimensional state and dominant frequency analysis plotted in (*h*_7_, *h*_8_) plane are displayed to show control results after adding four different stimulation plans. In particular, in Figures 5(a)–5(d), the white area and red area mean high saturated state and simple oscillation; the yellow region means SWD oscillation representing the pattern of absence seizures; the black area shows lowing firing state. Corresponding frequency analysis is displayed in Figures 5(e)–5(h), respectively.

In contrast to the original 42.3% size of SWD in Figure 4(a), SWD yellow areas, displayed in Figures 5(a)–5(d), have different extent shrink when applying stimulations to TRN. Most notably, an obvious reduction takes place after adding DBS, only 4.3% SWD size after adding DBS lead the pack followed by 18.8% SWD size after adding CBBP, 25.7% SWD size after adding CRS 1 : 0, and 36.3% SWD size after adding CRS 3 : 2. The four 2–4 Hz frequency domains of SWD are displayed in Figures 5(e)–5(h). By contrasting the size of SWD region in Figures 4(a) and 5(a)–5(d), Figures 5(a)–5(d) have four smaller SWD sizes. When the above four stimulations applied to TRN_*i*_, the activation level of TRN_*i*_ is suppressed under the effects of electrical stimulation, and activation level of SRN_*i*_ is gradually activated. The cortical firing states are transformed from SWD state to lowing firing state or simple oscillation state by excitatory effect from SRN_*i*_ on the cerebral cortex. Therefore, four stimulation plans are very effective for the inhibition of absence seizures. Owing to the size of SWD shrinked quite large, the treatment combinations of TRN_1_ and TRN_2_ under four plans can be adopted in inhibiting the pathological area of the CTMBC.

**Figure 6:**
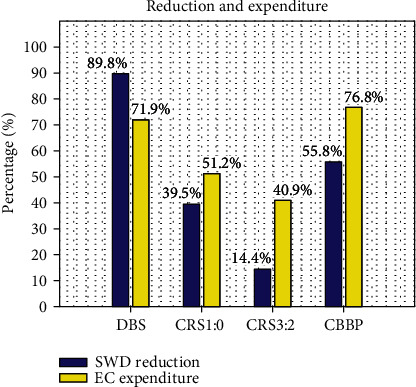
Comparison diagram in treatment effect and energy consumption of DBS, CBBP, CRS 1 : 0, and CRS 3 : 2 on the SWD in CTMBC. The blue bars and the yellow bars mean the SWD percentage reduction and the electric current (EC) expenditure after giving the four kinds of stimulation, respectively (color figure online).

In order to compare the best curative effect of the four stimulation plans, we measure two important indexes, SWD percentage reduction and electric current expenditure, displayed in Figure 6. The blue bars and the yellow bars mean the SWD percentage reduction and the electric current (EC) expenditure after giving the four kinds of stimulation, respectively. From Figure 6, the highest SWD reduction ratio was 89.8% in the treatment of DBS compared with other methods. Conversely, there is a reduction of only 14.4% under CRS3 : 2 stimulation. 55.8% SWD reduction under the CBBP stimulation and 39.5% under CRS1 : 0 stimulation rank in second and third place. On the other hand, the most and the least energy-efficient are 40.9% EC expenditure under CRS3 : 2 stimulation and 76.8% EC expenditure under CBBP stimulation, respectively; 51.2% EC expenditure under CRS1 : 0 stimulation and 71.9% EC expenditure under DBS stimulation rank in second and third place. However, the DBS and CBBP use relatively high stimulation current during the treatment that may have plenty of side effects on the brain. The continuous strong stimuli of DBS and CBBP might disrupt the nervous system. Although DBS and CBBP dramatically reduce the numbers of absence seizures, there are big risks in neurological complications and brain damage. Therefore, in this model, the CRS 1 : 0 characterized with comprehensiveness and high safety is best suited for epilepsy treatment.

## 4. Conclusions

Taking advantage of the coupled thalamocortical model and the bidirectional connection, we have investigated how the PY–TRN *h*_7_ pathway and the SRN–TRN *h*_8_ pathway induce the SWD firing of absence seizures. The four different states have proved that the appearance of state transitions, especially absence seizures in the CTMBC, originates from the change of the coupling strengths of *h*_7_ and *h*_8_ on TRN_1_. Combining with previous treatments, the TRN_1_ and the TRN_2_ are selected as a combined stimulation target in our research. Four different stimulation methods including DBS, CBBP, CRS 1 : 0, and CRS 3 : 2 are applied to the TRN_1_ and the TRN_2_ to explore the best cure by qualitatively comparing and analyzing. The CRS 1 : 0 reveals high cure rate and low risk for treating seizures in our model. However, the human brain has more intricate connections, more unknown factors, and huge difference than that of the theoretical CTMBC. But the insight of CTMBC may provide clinicians more therapeutic options in absence epilepsy patients. We will further investigate how electromagnetic radiation from memristor promotes the suppression of the SWD [43]. Ultimately, the new CTMBC model is presented in the paper to reproduce and control epileptic seizures by remodeling transmission strengths and connection configurations. Such obtained data might just change how we can explore the underlying outbreak range of the epilepsy and devise suitable neurological treatment schemes.

## Data Availability

The data used to support the findings of this study are included within the article.
